# Zhilong Huoxue Tongyu Capsules' Effects on ischemic stroke: An assessment using fecal 16S rRNA gene sequencing and untargeted serum metabolomics

**DOI:** 10.3389/fphar.2022.1052110

**Published:** 2022-11-18

**Authors:** Raoqiong Wang, Mengnan Liu, Guilin Ren, Gang Luo, Zhichuan Wang, Zhengxin Ge, Qingrong Pu, Wei Ren, Sijin Yang

**Affiliations:** ^1^ National Traditional Chinese Medicine Clinical Research Base, The Affiliated Traditional Chinese Medicine Hospital of Southwest Medical University, Luzhou, China; ^2^ Institute of Integrated Chinese and Western Medicine, Southwest Medical University, Luzhou, China

**Keywords:** ischemic stroke, intestinal flora, traditional Chinese medicine, ZhiLong HuoXue TongYu capsule, fecal microbial transplantation, untargeted serum metabolomics

## Abstract

Zhilong Huoxue Tongyu capsule (ZHTC) is an effective traditional Chinese medicine compound for the treatment of ischemic stroke, which is widely used in clinical ischemic stroke patients. However, it is uncertain whether ZHTC affects ischemic stroke through gut microbiota and serum metabolites. In this study, a rat model of middle cerebral artery occlusion (MCAO) was prepared. By evaluating motor nerve function score, cerebral infarct size, brain tissue damage and intestinal barrier damage, it was found that ZHTC improved stroke-related symptoms in MCAO rats. Using 16S rRNA gene sequencing, fecal microbial transplantation (FMT), untargeted metabolomics, and spearman correlation analysis of gut microbiota and serum metabolites, we found that ZHTC can regulate the abundance of p_Firmicutes, p_Bacteroidota,p_Proteobacteria, g_Prevotella, and g_*Lactobacillus*, and regulated 23 differential metabolites. Spearman correlation analysis found that Arginine was positively correlated with p_Firmicutes, o_Clostridiales, c_Clostridia, and negatively correlated with p_Bacteroidetes, c_Bacteroidia,o_Bacteroidales; L-Lysine was negatively correlated with f_Christensenellaceae; L-methionine was positively correlated with o_Lactobacillales, f_Lactobacillaceae, and g_*Lactobacillus*. Altogether, this study shows for the first time that ZHTC can ameliorate ischemic stroke by modulating gut microbiota and metabolic disturbances. This lays the foundation for further revealing the causal relationship between ZHTC, gut dysbiosis, plasma metabolite levels and ischemic stroke, and provides a scientific explanation for the ameliorating effect of ZHTC on ischemic stroke.

## Introduction

Stroke is divided into ischemic stroke and hemorrhagic stroke, of which ischemic stroke accounts for about 85% (Saini et al., 2021). Ischemic stroke (IS) is a cerebrovascular disease in which ischemia and blood reperfusion lead to brain tissue damage and cause changes in neurological function (Feske, 2021). It is one of the most important causes of neurological morbidity and mortality in the world. It imposes an enormous disease burden on patients and their families, and poses a serious threat to human health (Herpich and Rincon, 2020). Neuropathological changes in ischemic stroke include excitatory amino acid toxicity, calcium overload, oxidative stress injury, inflammatory response, and neuronal apoptosis (Paul and Candelario-Jalil, 2021). Clinicians generally use intravenous thrombolysis to treat ischemic stroke. However, its clinical application is limited due to the side effects of intracranial hemorrhage and reperfusion injury (Jolugbo and Ariëns, 2021). Therefore, there is an urgent need to develop new drugs for the treatment of Ischemic stroke. Recently, the role of intestinal flora and metabolomic analysis in ischemic stroke has received increasing attention.

There is two-way communication between the enteric nervous system and the central nervous system, which is called microbiota gut brain axis. Therefore, the enteric nervous system is called the human “second brain” (Pluta et al., 2021). Studies have shown that stroke can cause changes in the composition and function of intestinal flora. Once the intestinal microecosystem loses its stability, it can change the intestinal defense function and intestinal permeability, which will affect the central nervous system and adversely affect the prognosis of stroke (Hu et al., 2022). Gut microbiota can maintain body health by regulating the intestinal barrier (Takiishi et al., 2017; Di Tommaso et al., 2021). The expression of tight junction proteins zo-1, occludin-1 and claudin-5 decreased after ischemic stroke (Chen et al., 2019a). Ischemic stroke reduces the expression of intestinal mucosal tight junction protein, destroys intestinal barrier, increases intestinal permeability, and leads to the release of immune inflammatory factor (IL-1 β,IL-6,TNF- α). The proinflammatory cells is considered as a mediator of intestinal barrier dysfunction, such as intestinal permeability or incomplete barrier (Zhang P et al., 2021). Fecal microbiota transplantation (FMT) represents one of the most innovative treatments for gut microbiota modulation (Masucci and Quaranta, 2021). Benakis et al. (2016). found that FMT could reduce ischemic brain damage in mice by changing intestinal flora. It is an effective method to treat cerebral ischemic stroke by transplanting fecal bacteria rich in short chain fatty acids and supplementing butyric acid to interfere with intestinal microbiota (Chen et al., 2019b). In addition, stroke can lead to changes in a wide range of metabolites. Serum untargeted metabonomics has been used to identify the differential metabolites between the drug treatment group and the control group, which is helpful for screening biomarkers and studying the biological processes involved in differential metabolites (Au, 2018; Montaner et al., 2020). Therefore, studying the complex interaction between intestinal microbiota and metabolites in ischemic stroke provides a new perspective for the prevention, treatment and prognosis of ischemic stroke.

At present, the conventional treatment schemes for ischemic stroke include thrombolysis, conventional antiplatelet aggregation, and rehabilitation therapy (Rabinstein, 2020). However, many patients will suffer from limb weakness, language difficulties, anxiety, depression, and other sequelae, which seriously affect the quality of life of patients (Herpich and Rincon, 2020). Traditional Chinese medicine has formed a large number of famous and proven prescriptions in the long-term practice of treating ischemic stroke, and the effect is significant. Zhilong Huoxue Tongyu Capsule (ZHTC), a traditional Chinese medicine compound, has been used in the clinical treatment of ischemic stroke for more than 20 years. It has the effects of promoting Qi, dredging collaterals, promoting blood circulation, removing blood stasis and removing wind and phlegm. It has obtained the national patent (patent number:200810147774.1, drug batch number: 20210215, drug filing number: Z20200159000). ZHTC is composed of Hirudo nipponica Whitman [Hirudideae, the dried body of Hirudo nipponica Whitman], Pheretima aspergillum (E. Perrier) [Megascolecidae, the dried body of Pheretima aspergillum (E. Perrier)] , *Astragalus* membranaceus (Fisch.) Bge [Fabaceae, the dried root of *Astragalus* membranaceus (Fisch.) Bge], Cinnamomum cassia (L.) J. Pres [Lauraceae, the dried stem and twig of Cinnamomum cassia (L.) J. Pres] and Sargentodoxa cuneata (Oliv.) Rehder and E.H.Wilson [Lardizabalaceae, the dried stem and twig of Sargentodoxa cuneata (Oliv.) Rehder and E.H.Wilson], according to the ratio 0.32: 1.7: 2.3:0.86: 1.7. Hirudo nipponica Whitman is a traditional Chinese special medicinal aquatic animal that can repair the central nervous system by releasing neurotrophic substances (Boidin-Wichlacz et al., 2012). Hirudin is a promising early intervention drug for acute ischemic stroke. It can reduce acute ischemic stroke by inhibiting neuroinflammation mediated by NLRP3 inflammatory bodies (Li et al., 2022). Pheretima aspergillum (E. Perrier), an annelida, is a member of the giant earthworm family. It can prevent cerebral infarction and related cortical neuron death by reducing glial fibrillary acid (GFAP) and S100B astrocyte activation (Liu et al., 2012). *Astragalus* membranaceus (Fisch.) Bge is a legume of the genus *Astragalus*. Astragaloside IV is its main blood-activating component, which can alleviate cerebral ischemia-reperfusion injury by activating Nrf2 and inhibiting NLRP3 inflammasome-mediated pyroptosis inhibition (Xiao et al., 2021). Cinnamomum cassia (L.) is the dry bark of Cinnamomum cassia. Among them, cinnamaldehyde is a spice separated from cinnamon bark, which plays an anti-inflammatory role in various diseases. It alleviates atherosclerosis in mice by targeting the IκB/NF-κB signaling pathway (Li et al., 2019). The extracts and compounds of Sargentodoxa cuneata (Oliv.) Rehder&E.H. Wilson have a wide range of pharmacological activities, including anti-tumor, anti-inflammatory, antioxidant, antibacterial, anti sepsis, and anti arthritis effects, as well as the protective effects on cerebrovascular diseases (Zhang W et al., 2021).

The preliminary research team conducted quality control analysis on ZHTC through UPLC-HRMS. The results showed that ZHTC mainly included 8 compounds. They are L-epicatechin,calyx-7-O-β-glucoside, coumarin, red saponin, calyxine, cinnamaldehyde, formononetin, and wogonin (Mazhar et al., 2022). Based on previous studies, we selected 16srRNA gene sequencing and UHPLC-Q-TOF MS untargeted metabolomics to study the relationship between the gut microbiome and metabolic processes in ischemic stroke. Summarizing the mechanism of ZHTC on ischemic stroke, and it is hoped to provide reference for its further basic and clinical research.

## Materials and methods

### Laboratory animals

Male Sprague-Dawley rats (200–250 g) were purchased from the Experimental Animal Center of Southwest Medical University. All rats were housed in SPF animal rooms under standard conditions free of specific pathogens, light and dark cycles (Temperature 22°C ± 3°C, Humidity 50% ± 5%). The study was conducted in accordance with the guidelines published in the directive of the Council of the European Communities of 24 November 1986 (86/609/EEC). All animal procedures were approved by the Animal Ethics Committee of Southwest Medical University.

### Preparation of drugs

Hirudo nipponica Whitman, Pheretima aspergillum (E. Perrier) were soaked in 5 times the amount of 60% ethanol for 7 days, filtered it, and the filtrate was set aside. The dregs were mixed with the remaining three herbs and decocted twice with 7 times the amount of water, each time for 1 h. At the same time, collect the volatile oil: combine the decoction, filter it, concentrate the filtrate to the relative density of 1.05–1.10 (80°C), add ethanol to make the alcohol content reach 50%, leave it for 12 h and filter it; The liquid is combined with the alcohol extract, and the ethanol is recovered and concentrated it to a clear paste with a relative density of 1.20 (80°C); Take 200 g dextrin as the parent nucleus, make granules by boiling, crush into fine powder, spray the above volatile oil, mix well, and put into capsules.

### Experimental design

The rats were divided into five groups: Sham operation group (Sham group for short), Model group, High dose group of ZHTC (High dose group for short), Low dose group of ZHTC(Low dose group for short) and Nimodipine group. There are 10 rats in each group. According to the equivalent dose ratio converted from the body surface area of humans and rats, the doses required for each group of experimental rats were calculated as 1 g/Kg/d in the High dose group and 0.25 g/Kg/d in the Low dose group. According to the standard of equal volume of 3 ml, the concentration of the drugs in the High and Low dose groups was calculated by gavage. The Model group and the Sham group were given 3 ml of normal saline by gavage, and the Nimodipine group was given the standard of 6.25 mg/kg/d by gavage. Rats in all groups were given the corresponding drugs for 14 consecutive days, once a day in the morning by gavage.

### Establishment of MCAO model

A rat model of middle cerebral artery occlusion (MCAO) was prepared. After the anesthetized rats were intraperitoneally injected, they were fixed in the supine position, and a longitudinal incision was made at the midline of the neck to strip the subcutaneous muscle, and the left common carotid artery (CCA), external carotid artery (ECA), and internal carotid artery (ICA) were separated. The CCA and ECA were then ligated at the proximal end, and the proximal bifurcation at the distal end of the CCA was clamped. Then, a small incision was cut at the proximal end of the CCA from the bifurcation, and the suture was inserted into the CCA, and entered the ICA from the bifurcation of the blood vessel. The distal end of CCA was ligated from the bifurcation, and part of the suture was pulled out 2 h later for reperfusion. Rats in the Sham group were not inserted with sutures, and the rest of the steps were the same as those in other groups.

### Motor nerve function score

Zea Longa five point scoring method was used to evaluate motor nerve function defects caused by ischemic stroke. Scoring standard: 0 point: normal walking without any nerve defect; 1 point: the opposite front paw cannot be fully extended; 2 points: turn to the opposite side; 3 points: dump to the opposite side; 4 points: unable to walk spontaneously, loss of consciousness.

### Measurement of cerebral infarction area

In anesthetized rats, the whole brain was quickly taken, cut into 2 mm tissue sections, stained with 2% TTC for 5 min, and soaked in 4% paraformaldehyde for 6 h. Brain slices are arranged in order and photographed. Use ImageJ 1.41 software to calculate the cerebral infarction area (red area indicates no infarction; white area indicates infarction). The infarct area is calculated as the area of the non ischemic hemisphere minus the non infarct area of the ischemic hemisphere. Infarction volume = Infarction area × Thickness. The calculation formula of cerebral infarction percentage is as follows: cerebral infarction percentage = infarct volume/non ischemic hemisphere volume × 100%.

### HE staining

After anesthetizing the rats, open the chest to expose the heart, clamp the abdominal aorta with hemostatic forceps, cut a small opening of the right atrial appendage, insert a perfusion needle at the apex, and slowly inject normal saline until clear liquid flows out of the right atrial appendage. Replace it with 4% paraformaldehyde solution to continue perfusion. It can be seen that the whole body of the rats twitches and becomes stiff, then stop perfusion, and cut off the head to take the brain. Immerse in 4% paraformaldehyde solution for 24 h, dehydrate, embed in paraffin, slice, dewax and dye with hematoxylin (CR2109146, Wuhan, China) and eosin (CR2011064, Wuhan, China). The pathological morphology of the brain tissue was observed under a 400x light microscope. The ileum was dehydrated, embedded, sectioned, stained and sealed according to the SOP procedure for pathological examination. The sections were observed with a digital trinocular camera microscope (BA210 Digital, McAudi Industrial Group Co., Ltd.), and the area to be observed was selected to acquire 40x images and measured with Motic Images Advanced 3.2.

### Western blot analysis

Take out the colon tissue and put it into a 2 ml grinding tube, then add 3 mm steel beads and RIPA lysis solution (G2002-100ML, servicebio) to each tube, put it in a centrifuge (4°C, 12000 rpm, and 10 min), and take the supernatant after centrifugation. The protein concentration was determined with BCA protein quantification kit (P0009, beyotime). Then sample determination, protein denaturation, sample loading, electrophoresis, membrane transfer, blocking, incubation of antibodies and development and fixation are performed. Antibodies are as follows: Claudins-5 antibody (rabbit clone antibody, A10207, abclonal); Occludin-1 antibody (rabbit clone antibody, ab167161, Shanghai, China); ZO-1 antibody (rabbit clone antibody, AF5145, affinity); β- Actin antibody (rabbit clone antibody, AC026, abclonal).

### 16S rRNA gene sequencing

The fresh feces of rats in each group were collected aseptically. According to the experimental process, the total DNA of the microbiome was extracted, the target fragment was amplified by PCR, the amplification product was purified and recovered by magnetic beads, the amplification product was quantified by fluorescence, and the sequencing library was prepared. Then, the Illumina HiSeq platform was used for high-throughput sequencing.

#### Fecal microbial transplantation

FMT was performed according to an established protocol (Borody et al., 2013) .In FMT experiment, rats were divided into three groups: Model group, High dose group and FMT group. There are four rats in each group. FMT was started at the end of gavage in each group of rats. We gave the fecal supernatant of the High dose group to the Model group rats by tube feeding for FMT experiment. Briefly, fresh feces were collected from rats in the High dose group and put into 1 ml sterile PBS. They were immediately mixed in sterile water. The suspension was centrifuged at 2000 rpm for 10 min. Collect the supernatant and give it to the Model group rats by gavage, 200 μl per rat, once a day for 5 days. Evaluate and compare the stroke symptoms and intestinal flora results of rats in the Model group, High dose group and FMT group.

### Untargeted metabolomic analysis by UHPLC-Q-TOF MS

The metabolites in serum were detected by ultra-high performance liquid chromatography-tandem time-of-flight mass spectrometry (UHPLC-Q-TOF MS). The supernatant was injected for analysis. After separation with Agilent 1290 infinity LC ultra high performance liquid chromatography (UHPLC), mass spectrometry was performed with triple TOF 6600 mass spectrometer (AB SCIEX). The positive and negative ion modes of spray ionization (ESI) were used for detection. Then, XCMS software is used for peak alignment, retention time correction and peak area extraction. For the data extracted from XCMS, first identify the metabolite structure, preprocess the data, then evaluate the quality of the experimental data, and finally analyze the data.

### Statistical analysis

Statistical analysis was performed using SPSS 22.0 statistical software. The data were expressed as Mean ± SD. One-Way ANOVA test was used to compare the means among multiple groups, and LSD test was used for multiple comparisons after the event. When the test result was *p* < 0.05, the difference between groups was considered significant. Alpha diversity was determined by Chao1, Simpson and Shannon, and the significance of differences was verified by Kruskal-Wallis test and wilcoxon test; Beta diversity was visualized by PCoA, permutation multivariate analysis of variance (PERMANOVA) to assess differences in beta-diversity. LEfSe analysis identified microbial markers with differences in abundance between groups with LDA >2 and *p* < 0.05. OPLS-DA VIP>1 and *p* < 0.05 were the screening criteria for significantly different metabolites. Correlations between microbiota and metabolite data were calculated using the Spearman algorithm.

## Results

### ZHTC improves motor nerve function, reduces cerebral infarction area and brain tissue damage in MCAO rats

**FIGURE 1:**
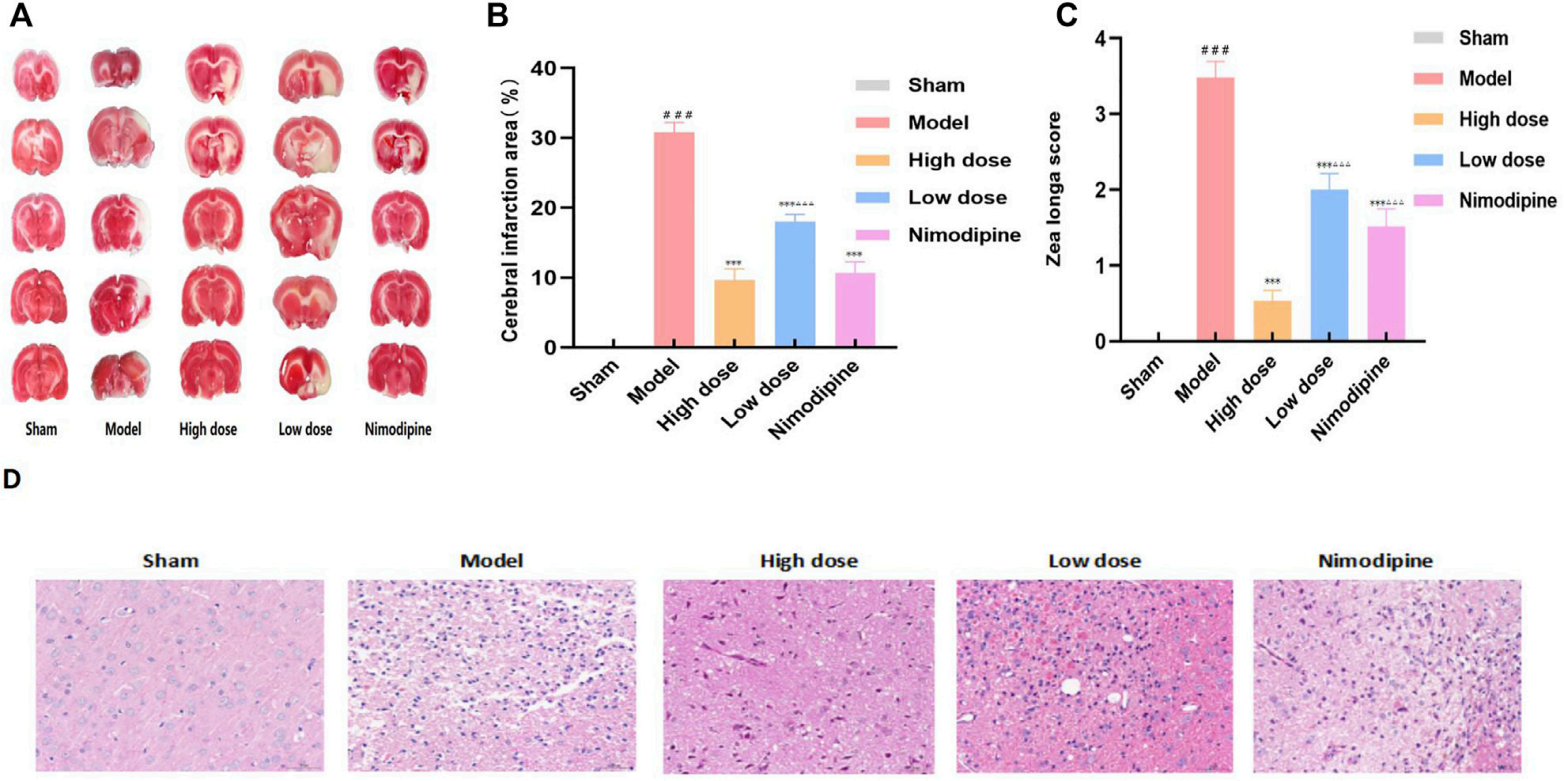
ZHTC improves motor nerve function, reduces cerebral infarction area and brain tissue damage in MCAO rats.

The efficacy of ZHTC in the treatment of ischemic stroke was assessed by measurement of cerebral infarct size (Figures 1A,B) and Zea Longa motor nerve function score (Figure 1C). Zea Longa score found that the score of the Model group was significantly higher than that of the Sham group. But after administration of ZHTC and nimodipine, the score was significantly reduced. And the score of the High dose group was lower than that of the Low dose group and Nimodipine group. It shows that ZHTC can improve the motor nerve function of MCAO rats, and the effect of high dose is better than that of low dose and nimodipine. The results of TTC staining showed that obvious edema and white infarct area were seen on the infarcted side of the Model group, indicating that the model was successfully established. After administration of ZHTC or nimodipine, the cerebral infarct size was significantly decreased, and the infarct size in the High dose group was smaller than that in the Low dose group.

In addition, brain tissue damage revealed by HE staining is shown in Figure 1D. Sham group: The pia mater structure of the brain tissue was intact, and there was no obvious inflammatory exudation; the neuronal cells in the brain were normal in shape, and there was no obvious degeneration and necrosis, inflammatory cell infiltration or gliosis. Model group: a small amount of lymphocytes can be seen in the connective tissue; large-scale necrosis of the hemisphere of the brain is more obvious, the necrotic area is lightly stained, necrotic neuron nuclei are pyknotic, lysed, nerve fibrinolysis, and a large number of glial cells proliferate, and the activated morphology can be seen Various microglia and astrocytes. High dose group, Low dose group and Nnimodipine group: the leptomeningeal structure of brain tissue is complete, and no obvious inflammatory exudation is found. Small area degeneration and necrosis of local brain tissue accompanied by proliferation of glial cells can be seen. Morphological structure of neurons is blurred and nerve fibers are dissolved. The results showed that ZHTC and Nimodipine can reduce brain tissue damage. Among them, compared with the Nimodipine group, the neuron morphological structure of the High dose group was closer to that of the Sham group, and the degree of brain tissue damage in the Nimodipine group was lower than that in the Low dose group.

The results of Zea Longa score, TTC staining and HE staining showed that ZHTC and Nimodipine could improve motor nerve function, reduces cerebral infarct area and brain tissue damage in MCAO rats, and ZHTC was superior to Nimodipine, and the higher the dose, the better the effect.

### ZHTC improves intestinal barrier damage in MCAO rats

**FIGURE 2:**
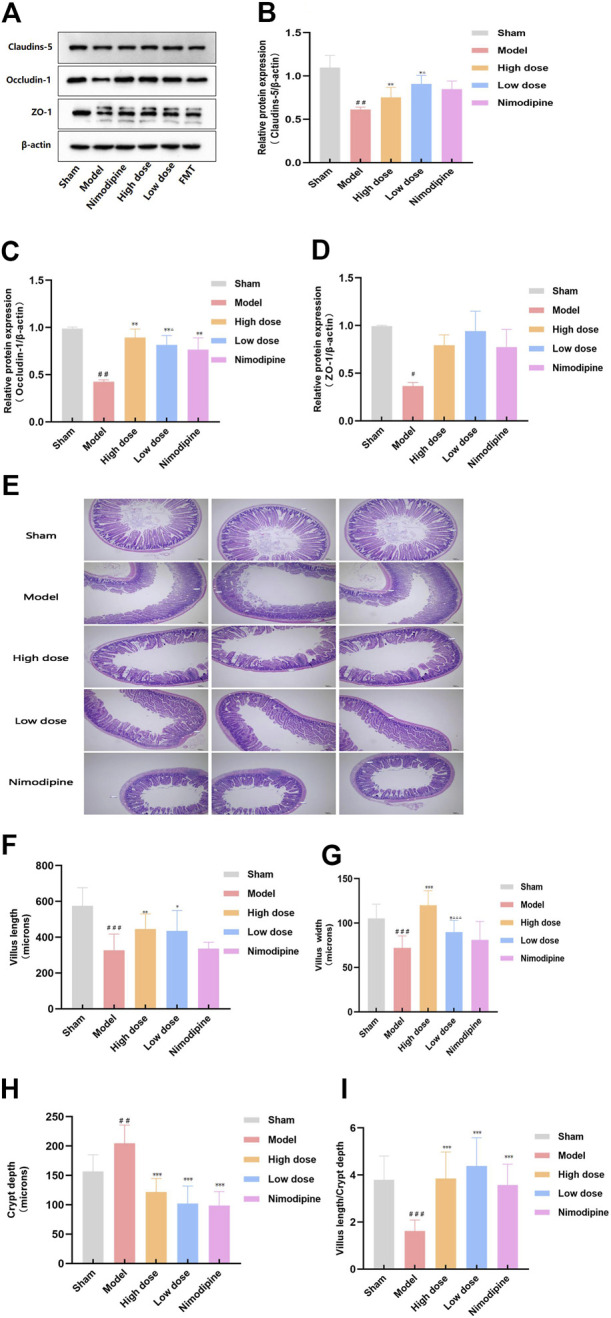
ZHTC improves intestinal barrier damage in MCAO rats.

Tight junction proteins connect successive epithelial layers, limiting the passage of cellular and intercellular nutrient molecules. It is an important component of the physiological barrier (Dokladny et al., 2016). In order to explore the effect of ZHTC on the destruction of intestinal barrier in MCAO rats, we studied the tight junction proteins claudin-5,occludin-1 and zo-1 (Figures 2A–D). The results showed that compared with the Sham group, the expression levels of claudin-5,cocludin-1 and zo-1 proteins in the Model group rats decreased, indicating that the intestinal barrier of ischemic stroke rats was damaged. the expression levels of occludin-1 and claudin-5 are increased by ZHTC treatment, and the effect of High dose group was better than that of Low dose group. But the expression levels of zo-1 has no obvious change. Nimodipine increased the expression levels of occludin-1 protein, but had no effect on claudins-5 and zo-1. In general, these findings indicate that nimodipine cannot completely reverse the destruction of the intestinal barrier. ZHTC treatment of ischemic stroke reverses the expression of tight junction proteins and improves the integrity of the intestinal barrier.

The epithelial layer, composed of villi and crypts, epithelium and endothelial cells, prevents the stimulation and invasion of external adverse factors. It plays a physical barrier role (Biomin Austria R&amp;D Headquarters, 2015). We further investigated villus length, villus width and crypt depth (Figures 2E–I). The results of this experiment showed that the length and width of villi in the ileum of the Model group were lower than those in the Sham group, and the depth of the crypt was higher than that in the Sham group. ZHTC increased villus length and width, decreased crypt depth, and increased the ratio of villus length to crypt depth. Nimodipine can reduce crypt depth, but has no significant effect on villus length and width. It shows that ischemic stroke can lead to damage to the intestinal barrier, and ZHTC has a proliferation and repair effect on the intestinal barrier.

### ZHTC improves intestinal flora imbalance in MCAO rats

**FIGURE 3:**
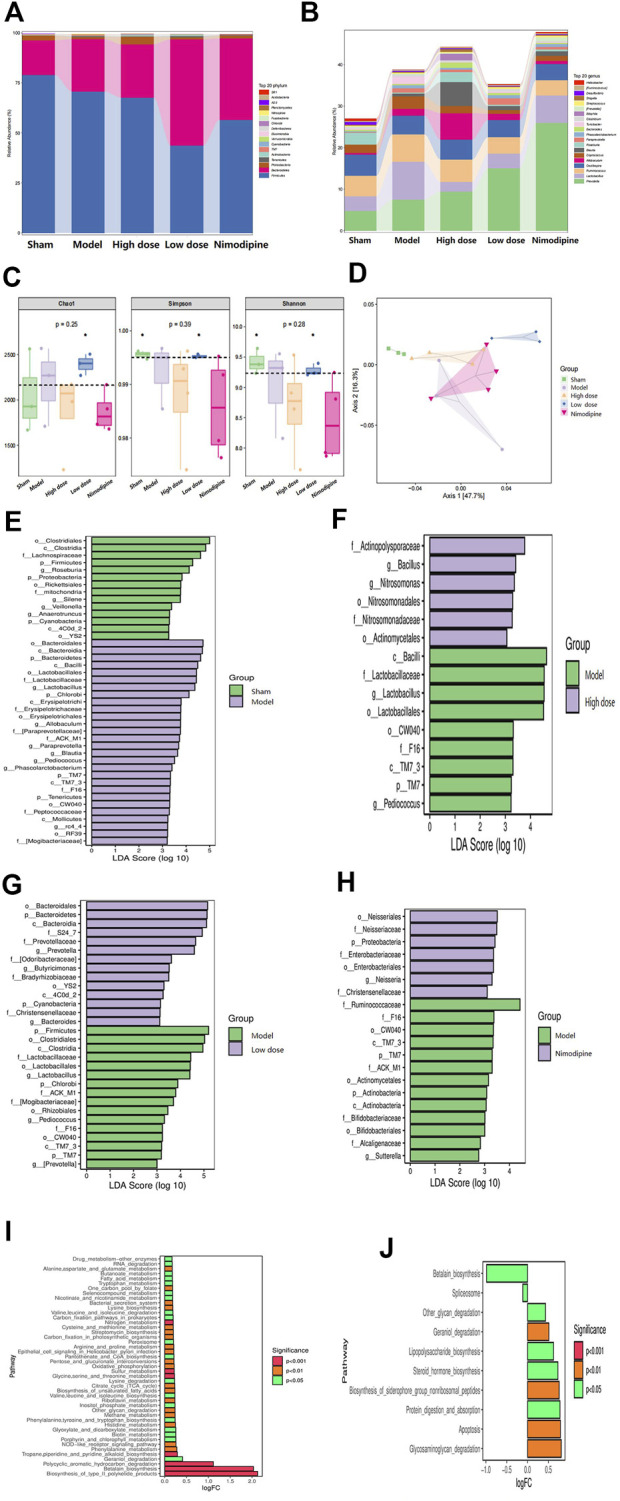
ZHTC improves intestinal flora imbalance in MCAO rats.

16S rRNA gene sequencing analysis was performed to evaluate the effect of ZHTC on gut microbiota dysbiosis in ischemic stroke rats. The multi-level analysis of intestinal microflora composition difference shows that at the Phylum level (Figure 3A), the abundance of p_ Firmicutes and p_ *Bacteroides* is the highest among the five groups, followed by p__ Proteobacteria. Among them, p__Proteobacteria decreased in the Model group, and increased in the High dose, Low dose and the Nimodipine group. Compared with the Model group, p__Firmicutes and p__Bacteroidota decreased in each administration group. At the genus level (Figure 3B), g_Prevotella and g_*Lactobacillus* were the most abundant among the five groups. The g_Prevotella in the Model group was significantly lower than that in the High dose, Low dose and Nimodipine groups; the number of g_*Lactobacillus* was significantly higher than that in the High dose, Low dose and Nnimodipine groups. It was found that p__ Firmicutes,p__ Bacteroidetes,g__ Prevotella,g__ Lachnospiraceae,g__ Ruminococcaeae was the dominant strain in the intestinal flora of five groups of rats, ranked by their relative abundance.

In order to comprehensively evaluate the alpha diversity of microbial communities, this study uses the Chao1 index to characterize the richness, and Simpson and Shannon indexes to characterize the diversity (Figure 3C). The results of Alpha diversity analysis indicated that the Low dose group could change the richness and diversity of intestinal flora. Changes in beta diversity were investigated using PCoA (Figure 3D). The study found that the Model group, the High dose group, and the Low dose group were clearly distinguished, except for the Nimodipine group, which was intersected with the Model group. The results showed that ZHTC significantly altered the beta diversity and composition of the gut microbiota. These data indicate that ZHTC can change the diversity and composition of intestinal flora in rats with ischemic stroke, but nimodipine has no such effect.

Next, to further investigate changes in gut microbiota biomarkers after administration, we performed LEfSe analysis to identify microbial markers (Figures 3E–H). Compared with the Sham group, the Model group are mainly enriched in o_Bacteroidales, c_Bacterodia, p_Bacteroidetes; Compared with the Model group, the High and Low dose groups were mainly enriched in f__Actinopolysporaceae, g__*Bacillus*, g__Nitrosomonas, o_Bacteroidales, p_Bacteroidetes, c_Bacterodia, and the Nimodipine group were mainly enriched in o__Neisseriales, f__Neisseriaceae, p_Proteobacteria.Potential functions of gut microbiota were analyzed with PICRUSt (Figures 3I,J). Compared with the Model group, ZHTC intervention changed arginine and proline metabolism, tryptophan metabolism and other metabolic pathways, valine, leucine and isoleucine biosynthesis, pentose phosphate pathway, RNA degradation, oxidative phosphorylation,etc.Studies have shown that ZHTC treatment leads to functional differences in gut microbiota in rats with ischemic stroke.

### FMT reduces stroke damage and alters intestinal flora

**FIGURE 4:**
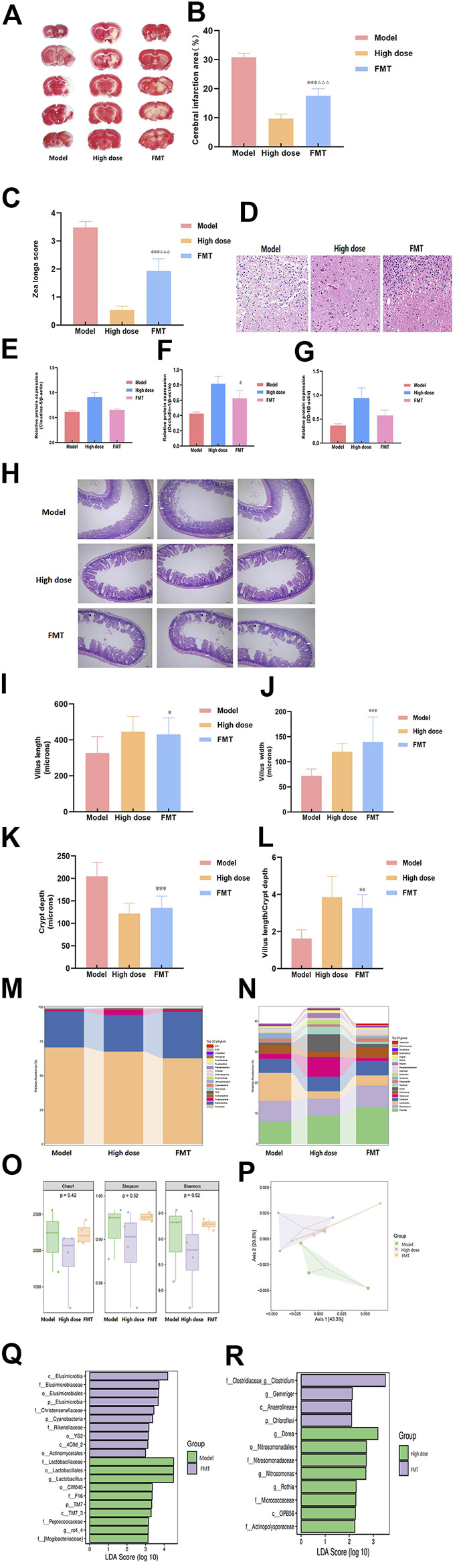
FMT reduces stroke damage and alters intestinal flora.

To further study the important role of gut microbiota in ischemic stroke, we administered the fecal supernatant of the High dose group to the Model group rats by gavage for FMT experiments. The results showed that FMT reduced cerebral infarction area (Figures 4A,B), the motor nerve function score (Figure 4C), and brain tissue damage (Figure 4D) of MCAO rats. It can increased the expression level of occludin-1, but it had no effect on claudin-5 and zo-1 (Figures 2A, 4E–G). And it increased villus length, villus width, villus length to crypt depth ratio, decreased crypt depth (Figures 4H–L). But the FMT group was not as effective as the High dose group.

The Model group, the High dose group and the FMT group were subjected to 16S rRNA gene sequencing analysis to evaluate the effect of FMT on the gut microbiota. The results showed that FMT altered the gut microbiota distribution in MCAO rats. Multilevel analysis of differences in gut microbiota composition found that at the phylum level (Figure 4M), the numbers of p__Bacteroidetes, p__Actinobacteria, and g__Prevotella in the FMT group were significantly higher than those in the Model group, and p__Firmicutes, p__Proteobacteria, and p__Tenericutes were lower than those in the Model group. At the genus level (Figure 4N), the numbers of g__Bacteroidetes, __Tenericutes, and g__Prevotella in the FMT group were significantly higher than those in the High dose group, and p__Proteobacteria, p__Actinobacteria, and p__Elusimicrobia were lower than those in the High dose group. FMT had no apparent effect on Alpha diversity (Figure 4O). PCoA showed that the FMT group and the Model group were significantly separated, and the FMT group was close to the High dose group (Figure 4P). In addition, LEfSe analysis (Figures 4Q,R) found that the FMT group was mainly enriched in c__Elusimicrobia, f__Elusimicrobiaceae, and o__Elusimicrobiales in comparison with the Model group. Compared with the High dose group, the FMT group was mainly enriched in f__Clostridiaceae_g_*Clostridium* and g__Gemmiger.

### ZHTC alters serum metabolites in MCAO rats

**FIGURE 5:**
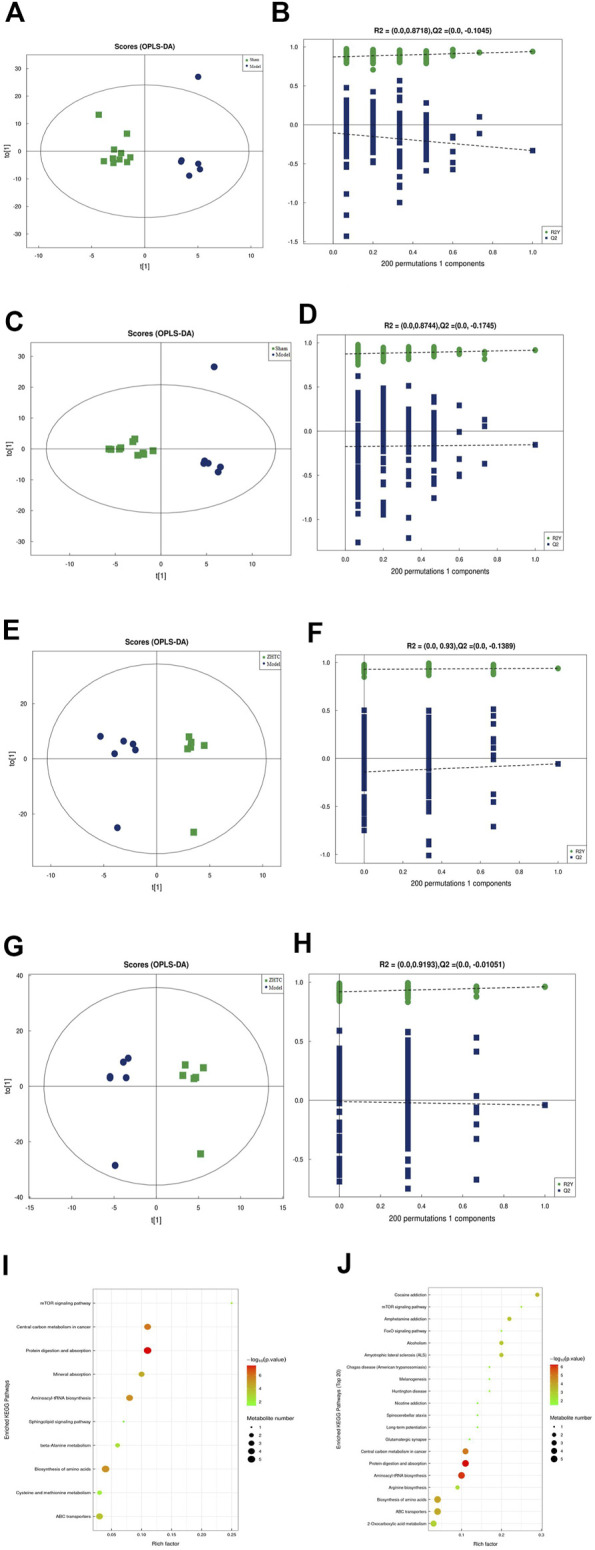
ZHTC alters serum metabolites in MCAO rats.

To explore the mechanism of action of ZHTC in improving MCAO rats, UHPLC-Q-TOF MS untargeted metabolomic analysis was performed. The score chart of OPLS-DA model (Figures 5A,C,E,G) shows that OPLS-DA model under positive and negative ion mode can distinguish between the Model group VS. the Sham group, the ZHTC Group VS. the Model group. In order to avoid overfitting of the supervised model during the modeling process, the permutation test was used to test the model to ensure the validity of the model. Figures 5B,D,F,H show the permutation test diagrams of the OPLS-DA model between the Model group VS. the Sham group, the ZHTC group VS. the Model group in the positive and negative ion mode. As the substitution retention gradually decreases, the R2 and Q2 of the random model gradually decrease. It can be seen from the result graph that the model does not have overfitting and the model is robust.

Using OPLS-DA VIP>1 and *p* < 0.05 as the screening criteria for significant differential metabolites, the differential metabolite molecules with biological significance were mined. We identified 14 differential metabolites in the Model group and the Sham group (Table 1), and 23 differential metabolites in the ZHTC group and the Model group (Table 2). The significance level of the metabolite enrichment of each pathway was analyzed and calculated by Fisher's Exact Test, so as to identify the metabolic and signal transduction pathways that were significantly affected (Figures 4I,J). The study found that there are important metabolic pathways in the Model group, including beta-Alanine metabolism, Cysteine and methionine metabolism, and Sphingolipid signaling pathway. The important metabolic pathways in ZHTC group are Arginine biosynthesis, Glutamatergic synapse, Arginine and proline metabolism, Tyrosine metabolism, D-Arginine and D-ornithine metabolism, Synaptic vesicle cycle, Dopaminergic synapse, D-Glutamine and D-glutamate metabolism.

**TABLE 1:** Differential metabolites in positive and negative ion patterns between the Model group and the Sham group.

ID	Adduct	Name	VIP	Fold change	p-value	m/z	rt(s)
M138T215	[M + H]+	Methylpicolinate	1.126035825	0.584089922	0.020169684	138.05375	214.6585
M177T78_2	[M + H]+	2-(2-butoxyethoxy)acetic acid	10.40437873	0.687599591	0.022493431	177.11126	77.953
M156T494	(M + H)+	L-Histidine	4.678609515	1.445188686	0.040103415	156.07588	493.671
M162T386	[M + H]+	L-carnitine	4.909370733	0.810106459	0.043214236	162.1122	385.647
M130T305	(M-H)-	L-Leucine	1.872446805	0.656171012	0.000543967	130.08626	304.721
M104T364	[M-H]-	Serine	1.551277077	1.160950138	0.007854412	104.03456	363.526
M480T76	[M-H-H2O]-	Taurodeoxycholic acid	1.594936646	1.966386296	0.010602133	480.2759	75.804
M88T336	[M-H]-	Beta-alanine	2.612304533	1.272364957	0.012993054	88.03979	336.18
M699T104	[M-H]-	1,2-dioleoyl-sn-glycero-3-phosphate	1.426782481	2.453506983	0.029980683	699.49341	103.928
M200T300	[M-H]-	Cysteine-s-sulfate	1.862854276	0.625368266	0.032806296	199.96797	299.765
M195T488	(M-H)-	Galactonic acid	2.045106902	3.597894032	0.033654246	195.04992	488.3885
M114T304_2	[M-H]-	Proline	6.985482267	1.296198708	0.042264815	114.05627	304.224
M135T103	[M-H]-	Phenylacetic acid	1.970680153	1.251076267	0.048289503	135.04413	103.071
M867T78	[M + Hac-H]-	Pc(18:1e/8-hepe)	1.538903207	2.479218415	0.049929666	866.58799	78.1255

**TABLE 2:** Differential metabolites in positive and negative ion patterns. Between the ZHTC group and the Model group.

ID	Adduct	Name	VIP	Fold change	p-value	m/z	rt(s)
M177T78_2	[M + H]+	2-(2-butoxyethoxy)acetic acid	14.09333699	1.735371217	0.001482418	177.11126	77.953
M182T296	[M + H]+	DL-tyrosine	1.411103804	0.593404096	0.008475857	182.07936	296.34
M191T246	[M + H]+	Thr-Ala	1.213068651	0.780781616	0.015241223	191.08379	246.391
M378T289	(M + K)+	Papaverine	1.14782005	0.338891582	0.017422822	378.11361	288.733
M229T416	[M + H]+	Pro-hyp	2.497204792	0.699251486	0.018594187	229.11674	415.85
M274T37	[M + H]+	Fenpropidin	1.767242766	0.893088617	0.019248734	274.27249	36.62
M527T406	[M + Na]+	Maltotriose	1.135324879	3.204002338	0.022313051	527.15528	406.0625
M160T380	[M + H]+	5-aminovaleric acid betaine	7.554715269	0.61314446	0.022542778	160.13251	380.251
M188T480	(M + CH3CN + H)+	L-Lysine	4.26060428	0.363105367	0.023477904	188.1388	480.451
M134T25	[M + H-CH6O2]+	Dl-normetanephrine	1.904441051	0.594073361	0.026849344	134.05913	24.861
M175T430	[M + H]+	Arginine	10.16015436	0.667409252	0.047716041	175.11843	430.205
M146T387_2	[M-H]-	Glutamic acid	4.576138307	0.731443932	0.006011208	146.04573	386.5205
M181T200	[M-H]-	Hydroxyphenyllactic acid	2.263119292	0.673335627	0.00796875	181.04962	199.984
M274T476	[M-H]-	Glu-Lys	1.529947355	0.641568539	0.009022066	274.13946	476.291
M195T488	(M-H)-	Galactonic acid	1.339466965	0.605591782	0.009679995	195.04992	488.3885
M269T99_1	[M-H-CO2]-	Loxistatin acid	1.107459112	0.677916838	0.0185072	269.17421	99.389
M157T186_2	[M-H]-	Allantoin	7.110834765	0.759664588	0.027575228	157.03636	186.246
M251T181	[M-H]-	Deoxyinosine	1.220224189	0.314563244	0.028679276	251.07707	181.168
M180T295_2	[M-H-C2H2O]-	N-acetyl-l-tyrosine	6.626553136	0.716154383	0.031308886	180.06656	295.174
M218T266	[M-H]-	Pantothenate	3.232204552	0.770877421	0.033380317	218.10263	265.553
M148T280	[M-H]-	L-methionine	1.687557909	0.819411472	0.039099925	148.04274	279.978
M153T29	[M-H]-	beta.-resorcylic acid	1.398358525	0.790192058	0.0415821	153.01849	28.671
M190T144_2	(M-H)-	5-Hydroxyindoleacetate	1.701088338	1.981895555	0.047575883	190.04934	144.433

### Spearman correlation analysis of microbial diversity and serum metabolites

**FIGURE 6:**
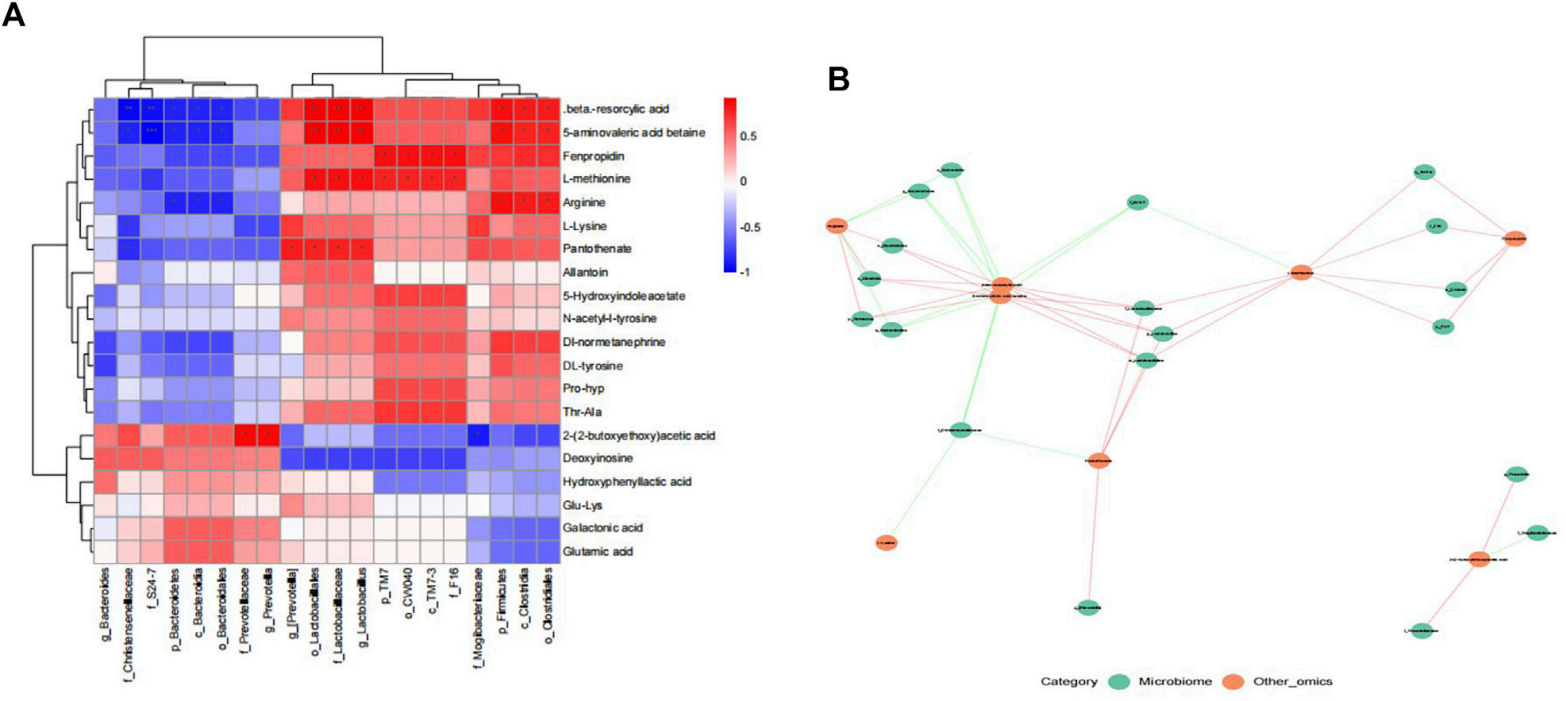
Spearman correlation analysis of microbial diversity and serum metabolites.

To evaluate the interaction between gut microbiota and metabolites, We conducted Spearman correlation analysis on the differential bacteria and metabolites between ZHTC group and model group. The study found significant correlations between most serum metabolites and gut microbiota (Figures 6A,B). Among them, Arginine is positively correlated with p_Firmicutes, o_Clostridiales, c_Clostridia, and negatively correlated with p_Bacteroidetes, c_Bacteroidia, o_Bacteroidales; L-Lysine is negatively correlated with f_Christensenellaceae; L-methionine is positively correlated with o_Lactobacillales, f_Lactobacillaceae, and g_*Lactobacillus*.The results showed that the improvement of ZHTC on ischemic stroke was related to the changes of intestinal flora and its metabolites.

## Discussion

In this study, we found that the MCAO model was successfully established by Zea Longa motor nerve function score and TTC staining. After ZHTC treatment, the motor nerve function was significantly improved, and the area of cerebral infarction and brain tissue damage were significantly reduced. It shows that ZHTC can effectively improve the symptoms of ischemic stroke. The gut barrier is the link between the gut microbiome and the brain (Trzeciak and Herbet, 2021). To maintain the structural and functional integrity of the intestinal barrier, the expression of tight junction proteins such as claudin-5, occludin-1 and zo-1 in the intestinal tissue plays an important role (Xue et al., 2018). The detection of the expression level of tight junction protein can not only reflect the damage of the intestinal barrier, but also reflect the recovery degree of the intestinal barrier. The increased expression of claudin protein can inhibit the adhesion of bacteria on the intestinal mucosa, prevent the increase of epithelial cell permeability, and protect the intestinal mucosal barrier function (He et al., 2019; Wang et al., 2019). This study found that the intestinal barrier was disrupted in ischemic stroke rats. Ococcludin-1, claudin-5 were recovered by ZHTC treatment. It is suggested that ZHTC improves intestinal barrier integrity by increasing the expression level of tight junction proteins. In addition, the small intestinal villi are the barrier of the small intestinal wall. The villous epithelial cells are in a state of continuous renewal. Under normal conditions, they have a strong ability to proliferate and repair. They can be repaired within 24 h of damage to maintain the normal tissue structure of the villi (Rui et al., 2018; Luo et al., 2019; Yin et al., 2019). The length and width of villi can be used to judge the damage degree and proliferation and repair ability of intestinal mucosa. The greater the villus length and width, the smaller the intestinal mucosa damage (Gan, 2011; Ha and Ao, 2011). The intestinal crypt is a tubular gland formed by the intestinal epithelium sinking to the lamina propria at the root of villi, which opens between adjacent villi. The increase of crypt depth will weaken the intestinal mucosal repair ability and cannot prevent harmful bacteria from colonizing the intestine (Chen et al., 2021; Kan et al., 2022). Therefore, the higher the ratio of villus length to crypt depth, the better the protection of intestinal mucosa. Our findings suggest that ischemic stroke can lead to intestinal mucosal damage by decreasing villus length, villus width, and the ratio of villus length to crypt depth and increasing crypt depth. However, ZHTC has a proliferative and repairing effect on the intestinal mucosa by reversing the above results. These results suggest that ZHTC can improve stroke symptoms and intestinal barrier in ischemic stroke rats.

Based on the improvement effect of ZHTC on stroke symptoms and intestinal barrier in MCAO rats, we investigated the effect of ZHTC on the intestinal flora of ischemic stroke. In this study, we found that ZHTC can alleviate ischemic stroke by modulating the composition of the gut microbiota. At the phylum level, it regulates the abundance of Firmicutes, Bacteroidota, and Proteobacteria. At the genus level, it can regulate the abundance of Prevotella and *Lactobacillus*. The human intestinal flora is mainly composed of Firmicutes and Bacteroidetes, and the rest are mostly Actinobacteria, Proteobacteria and Verrucomicrobia (Eckburg et al., 2005). The hallmarks of dysbiosis after stroke are changes in bacteria such as Firmicutes, Bacteroidetes, and Actinobacteria (Sorboni et al., 2022). Spychala et al. (2018) found dysbiosis in the gut microbiota in mice after stroke, resulting in an increased ratio of Firmicutes to Bacteroidota. Another study using the MCAO mouse stroke model found that 3 days after severe stroke, the gut microbiota composition changed significantly, mainly manifested by reduced species diversity and overgrowth of Bacteroidetes (Singh et al., 2016). After stroke, the intestinal flora of patients with cerebral infarction changed significantly, with a significant increase in opportunistic pathogenic bacteria in the intestine, such as Enterobacteriaceae, Veronicaceae and Streptococcaceae, and a significant decrease in resident bacteria, such as Prevotella and *Bacteroides* (You et al., 2018). A study of 93 patients with ischemic stroke showed that the abundance of Prevotella and *Lactobacillus* was associated with cognitive impairment after stroke (Ling et al., 2020). Yamashiro et al. assessed the fecal microbiota and fecal organic acids in stroke patients, they found that ischemic stroke was associated with a reduced number of *Lactobacillus* (Yamashiro et al., 2017). It is worth noting that our results also found that Firmicutes, Bacteroidetes, Prevotella, Lachnospiraceae, and Ruminococcaceae were the dominant strains in the intestinal flora of five groups of rats, and ZHTC could reverse the above-mentioned intestinal flora of MCAO rats. After FMT, we found that the FMT group had the same effect as the High dose group. It showed significantly better performance in motor nerve function score, and smaller cerebral infarction volume and brain tissue damage than the Model group. Interestingly, compared to the Model group, the rats in the FMT group reduced the damage of intestinal barrier and changed the intestinal microbiota. These findings indicate that FMT therapy is effective in normalizing the changes of microbial communities caused by stroke, which may play a neuroprotective role after stroke. And it was demonstrated that the protective effect of ZHTC in rats with ischemic stroke was mediated through intestinal flora.

After demonstrating the role of ZHTC in improving ischemic stroke prognosis and modulating gut microbiota disturbance, we performed an untargeted metabolomic study of its underlying mechanism. A total of 23 differential metabolites were altered by ZHTC treatment. In this study, Arginine was positively correlated with p_Firmicutes, o_Clostridiales, c_Clostridia, and negatively correlated with p_Bacteroidetes, c_Bacteroidia, o_Bacteroidales.Arginine, a non-essential amino acid, has been reported to improve cerebral blood flow and metabolism and reduce infarct volume in rats with cerebral ischemia (He et al., 1995; Temiz et al., 2003). And it has neuroprotective effects by inhibiting HIF-1α/LDHA-mediated inflammatory response after cerebral ischemia/reperfusion injury (Chen et al., 2020). Arginine supplementation improves gut injury and gut immunity. Arginine accelerates gut health through cytokines and gut microbiota, reduces the effect of gut Firmicutes on glycolipid metabolism, and in turn alters gut microbial composition (Wu H et al., 2020; Xie et al., 2020). In addition,L-Lysine was negatively correlated with f_Christensenellaceae.Lysine is a basic amino acid. Histone H3 crotonylation at lysine 18 is a surprisingly abundant modification in the small intestinal crypts and colon, and microbiota-derived short-chain fatty acids promote histone crotonylation in the colon *via* histone deacetylases (Fellows et al., 2018). Interestingly, oral administration of lysine and arginine was found to be neuroprotective against cerebral infarction induced by transient middle cerebral artery occlusion/reperfusion in rats (Kondoh et al., 2010).L-methionine was positively correlated with o_Lactobacillales, f_Lactobacillaceae, g_*Lactobacillus*.It was found that methionine can improve the acid resistance and nisin production of L. lactis F44 by regulating cell wall remodeling (Wu M et al., 2020). S-adenosyl-L-methionine has a beneficial effect on the outcome of ischemic injury by reducing the blood-brain barrier breakdown and neuronal death (Rao et al., 1997). It can also reduce the oxidative stress damage to the brain of rats with permanent focal ischemia and global cerebral ischemia-reperfusion (Villalobos et al., 2000). Our study indicated that Arginine, L-Lysine and L-methionine may be important biomarkers for ischemic stroke. Their metabolism in stroke and their relationship with the gut microbiota set the stage for our next steps.

## Conclusion

In conclusion, our study preliminarily confirmed that ZHTC can improve ischemic stroke, and this mechanism is related to maintaining intestinal microecological homeostasis and regulating metabolic disorder. It provides a certain degree of scientific basis for the clinical application of ZHTC in the treatment of ischemic stroke, and it also lays a foundation for further elucidating its potential mechanism.

## Data Availability

The datasets presented in this study can be found in online repositories. The names of the repository/repositories and accession number(s) can be found below: NCBI BioProject (https://www.ncbi.nlm.nih.gov/bioproject), PRJNA891433.

## Abbreviations

ZHTC, Zhilong Huoxue Tongyu capsule; MCAO, middle cerebral artery occlusion; FMT, fecal microbial transplantation; LDA, Effect Size Analysis; LEfSe Analysis.
